# The Mediating Role of Interpersonal Needs in Perceived Parenting Styles and Social Media Addiction Among University Students: Cross-Sectional Study

**DOI:** 10.2196/91861

**Published:** 2026-05-27

**Authors:** Yujie Liu, Suping Wang, Ting Wei, Birong Wu, Xin Ge, Shangbin Liu, Chen Xu, Shunyu Tao, Xiaohong Fan, Fan Hu, Ying Wang, Xue Yang, Yong Cai

**Affiliations:** 1 Tongji Hospital, School of Medicine, Tongji University, Shanghai, China; 2 Institute of Clinical Research, Tongren Hospital, Shanghai Jiao Tong University School of Medicine, Shanghai, China, 86 021-52039999; 3 Shanghai Jiao Tong University School of Medicine, Shanghai, China; 4 Shanghai Public Health Clinical Center, Shanghai, China; 5 JC School of Public Health and Primary Care, Faculty of Medicine, The Chinese University of Hong Kong, Hong Kong; 6 Center for Community Health Care, Shanghai Jiao Tong University China Hospital Development Institute, Shanghai, China

**Keywords:** social media addiction, parenting styles, university students, unmet interpersonal needs, psychological mediation, latent profile analysis

## Abstract

**Background:**

The rapid development of digital technologies has intensified concerns about social media addiction, particularly among university students. The perceived parenting styles they experienced may influence this risk, with unmet interpersonal needs acting as potential mediators.

**Objective:**

This study aimed to investigate whether perceived burdensomeness and thwarted belongingness mediate the relationship between perceived parenting and social media addiction among university students.

**Methods:**

A cross-sectional survey was conducted among 1766 university students from March 2023 to May 2023. Parenting styles were assessed using the short-form Egna Minnen av Barndoms Uppfostran (Swedish for “My memories of upbringing”) for Chinese, unmet interpersonal needs were assessed via the 15-item version of the Interpersonal Needs Questionnaire, and social media addiction was assessed through the Social Network Addiction Tendency Scale. Parenting profiles were identified using latent profile analysis, and mediation models were examined through path analysis.

**Results:**

Three perceived parenting profiles were identified: supportive (1094/1766, 61.9%), emotionally distant (192/1766, 10.9%), and controlling-critical (480/1766, 27.2%). Path analyses indicated indirect effects of parenting profile on social media addiction via unmet interpersonal needs. Compared to the supportive group, the emotionally distant (β=0.107 for perceived burdensomeness; β=0.354 for thwarted belongingness) and controlling-critical (β=0.543 for perceived burdensomeness; β=0.457 for thwarted belongingness) groups reported higher unmet interpersonal needs, which in turn were positively associated with social media addiction (β=0.119 for perceived burdensomeness; β=0.158 for thwarted belongingness). Multigroup analyses further showed that perceived burdensomeness was significantly associated with social media addiction among male students (β=0.180; *P*<.001) but not among female students (β=0.038; *P*=.30).

**Conclusions:**

Unmet interpersonal needs mediate the link between maladaptive parenting and social media addiction. Early interventions that promote emotional warmth and reduce rejecting and controlling behaviors may help prevent digital dependence among young adults, particularly when tailored to specific interpersonal vulnerabilities.

## Introduction

The rapid proliferation of digital technologies has reshaped human interaction, offering unprecedented opportunities for communication. In China, this shift has been accompanied by the widespread use of locally dominant social media platforms such as WeChat and Weibo, particularly among university students. As these technologies have become increasingly embedded in everyday life, concerns about digital dependence have also grown. Social media addiction is a specific manifestation of digital dependence characterized by impaired control over social media use, obsessive preoccupation with online activities, difficulty resisting the urge to engage, and a preference for online over face-to-face interactions [1]. A meta-analysis showed that the pooled prevalence of social media addiction reached 24% across 32 countries, with higher rates observed among young people [2]. University students are particularly vulnerable due to unlimited internet access, lack of parental supervision, flexible schedules, and developmental demands for social interaction [3]. In this population, social media addiction has been linked to adverse consequences, including poor sleep quality, reduced academic performance, and increased risks of mental health problems [3-5]. These concerns underscore the importance of clarifying the pathways underlying the development of social media addiction.

Although numerous studies have identified the roles of proximal individual factors such as attachment styles and personality traits in social media addiction [6,7], its development may also be shaped by broader environmental influences. Early family experiences are particularly crucial in shaping psychological vulnerabilities related to social media addiction [8]. Against the backdrop of rapid digital change, traditional family expectations and relational norms may remain influential [9], whereas young adults are increasingly embedded in highly digitalized social environments. In this context, the relationship between parenting and online engagement warrants particular attention. Previous research among Chinese university students has shown that poor family functioning is associated with higher levels of social networking addiction [10]. Maladaptive parenting practices such as overprotection and psychological control have been positively associated with problematic social media use [11,12], whereas positive parenting may attenuate the association between negative life events and social media addiction [13]. However, the mechanisms through which parenting style influences social media addiction remain poorly understood.

According to the social compensation hypothesis, individuals facing difficulties in offline social interactions may turn to online platforms as a compensatory strategy [14]. Unsatisfied interpersonal needs and social deficits can increase the propensity for excessive use, which may develop into an addictive pattern sustained by negative reinforcement [15]. Thwarted belongingness and perceived burdensomeness represent 2 fundamental unmet interpersonal needs, which are conceptualized within the framework of the interpersonal theory of suicide as critical factors contributing to adverse psychological states [16]. Thwarted belongingness reflects the perception that one’s need for social connection is unfulfilled, whereas perceived burdensomeness represents the belief that one is a burden to others. Both conditions have been linked to a wide range of adverse health outcomes, including a higher risk of suicide [17-19]. In collectivist cultural contexts such as China, these interpersonal needs may be particularly relevant given the strong emphasis on relational interdependence and social harmony [20]. There is evidence from Chinese samples further suggesting that individuals experiencing these unmet interpersonal needs may engage in excessive social media use to seek social connection [21], supporting the compensatory pathway toward social media addiction. Moreover, parenting practices such as low emotional warmth and high verbal hostility have been shown to predict the development of unmet interpersonal needs in offspring [22]. These findings highlight thwarted belongingness and perceived burdensomeness as potential pathways linking parenting styles to social media addiction.

Despite the theoretical plausibility of these pathways, empirical evidence examining the mediating effects of thwarted belongingness and perceived burdensomeness remains limited. To address this gap, this study investigates whether these 2 dimensions of unmet interpersonal needs mediate the relationship between perceived parenting styles and social media addiction among Chinese university students. Clarifying these mechanisms can advance understanding of the psychological processes underlying social media addiction and inform the development of targeted, evidence-based interventions for digitally vulnerable emerging adults.

## Methods

### Participants and Procedure

A cross-sectional survey was conducted between March 2023 and May 2023 in Shanghai, China. Participants were recruited using a multistage sampling method. In the first stage, universities were selected based on type, including 2 medical universities, 2 liberal arts universities, and 1 science and engineering university. In the subsequent stages, participants were further stratified by academic year and major for sampling purposes.

Electronic questionnaires were administered via the Wenjuanxing platform, a professional online survey system commonly used in academic research in China. The questionnaire was designed with mandatory responses for all items and embedded logical validation rules to ensure data quality. Only fully completed questionnaires could be submitted successfully.

Of the 2125 questionnaires distributed, 1768 (83.2%) were returned. Of these 1768 questionnaires, after excluding invalid responses (eg, identical answers across items or completion times under 5 minutes), 1766 (99.9%) valid questionnaires were retained for analysis.

### Measurements

#### Perceived Parenting Style

Perceived parenting style was assessed using the short-form Egna Minnen av Barndoms Uppfostran for Chinese (S-EMBU-C) [23], adapted from the original self-reported Egna Minnen av Barndoms Uppfostran (Swedish for “My memories of upbringing”) [24]. The S-EMBU-C includes 42 items organized into 2 parallel scales for fathers and mothers. Each scale comprises 21 items measuring 3 dimensions: rejection (eg, “My father/mother treat me in such a way that I feel ashamed”), emotional warmth (eg, “My father/mother try to encourage me to become the best”), and overprotection (eg, “My father/mother get overly anxious that something might happen to me”). Each item is rated on a 4-point Likert scale ranging from 1 (“never”) to 4 (“always”). The S-EMBU-C has demonstrated good reliability and validity among Chinese university students [23]. In this study, Cronbach α values were 0.949 to 0.953 for rejection, 0.949 to 0.951 for emotional warmth, and 0.778 to 0.800 for overprotection.

#### Interpersonal Needs

Interpersonal needs were assessed using the 15-item version of the Interpersonal Needs Questionnaire (INQ-15), which is a shortened form of the original scale [25]. The INQ-15 captures 2 dimensions: perceived burdensomeness and thwarted belongingness, which have been validated in prior research as key indicators of suicide-related interpersonal needs [26,27]. Each item is rated on a 7-point Likert scale ranging from 1 (“not true for me at all”) to 7 (“very true for me”), with higher total values reflecting greater unmet interpersonal needs. The Chinese version of the INQ-15 has shown satisfactory reliability and validity in Chinese university student samples [28]. In this study, Cronbach α values were 0.974 for perceived burdensomeness and 0.872 for thwarted belongingness.

#### Social Media Addiction

Social media addiction was assessed using the Social Network Addiction Tendency Scale [29]. The scale includes 8 items, each rated on a 5-point Likert scale from 1 (“strongly disagree”) to 5 (“strongly agree”). All items are positively scored (eg, “Using social networks makes it hard for me to focus on my studies”), with higher total scores indicating a greater propensity toward social media addiction. Previous studies have demonstrated that this scale possesses good psychometric properties among Chinese university students [29]. In this study, the scale showed good internal consistency, with a Cronbach α coefficient of 0.910.

#### Covariates

Covariates included sex (male and female), age, academic year (first year, second year, third year, and fourth year or above), university type (medical, liberal arts, and science and engineering), only child status (yes or no), and parental educational levels (primary school or lower, junior or senior high school, and college or higher).

### Statistical Analysis

Descriptive analyses were conducted to summarize participants’ demographic characteristics and the distribution of social media addiction. Group differences in social media addiction across demographic groups were examined using 2-tailed independent-sample *t* tests or one-way ANOVA. Pearson correlations were computed to assess the associations among parenting styles, perceived burdensomeness, thwarted belongingness, and social media addiction.

Latent profile analysis (LPA) was performed using the R package *tidyLPA* (R Foundation for Statistical Computing) to identify distinct parenting profiles based on paternal and maternal rejection, emotional warmth, and overprotection. Models with 2 to 5 classes were estimated. Model fit was evaluated using the Akaike information criterion, Bayesian information criterion (BIC), sample size–adjusted BIC, entropy, and bootstrap likelihood ratio test. Class proportions and interpretability were also taken into account.

Hierarchical regression analyses were then conducted to examine the association between parenting profiles and social media addiction. In model 1, only parenting profiles were included. Model 2 further adjusted for demographic covariates (sex, age, university type, academic year, only child status, and parental educational levels). Model 3 additionally incorporated perceived burdensomeness and thwarted belongingness.

Finally, path analysis was used to test the pathways from parenting profiles to social media addiction via perceived burdensomeness and thwarted belongingness. Parenting profiles were dummy coded, with the largest group serving as the reference category. Model fit was assessed using the comparative fit index, Tucker-Lewis index, root mean squared error of approximation, and standardized root mean squared residual. Direct and indirect effects were tested using bootstrapping with 5000 resamples.

### Ethical Considerations

All procedures performed involving human participants were in accordance with the 1964 Declaration of Helsinki. The study protocol was approved by the ethics committee of Shanghai Jiao Tong University School of Medicine (SJUPN-201813). Before accessing the survey, participants were required to read and electronically sign an informed consent form explaining the study’s purpose, voluntary participation, and confidentiality. Participants received a CN ¥5 (approximately US $0.74) digital incentive after completing the survey.

## Results

### Descriptive Statistics and Correlations

Table 1 presents the demographic characteristics of the 1766 participants. The sample included 48.5% (n=857) men and 51.5% (n=909) women, with a mean age of 19.9 (SD 1.5) years. Regarding university type, 44.9% (n=793) of the participants were from medical universities, 14.3% (n=252) were from the science and engineering university, and 40.8% (n=721) were from liberal arts universities. Approximately half (n=895, 50.7%) were first-year students. Social media addiction scores did not significantly differ by gender, age, or academic year. However, significant differences were observed across university types (*P*<.001), with students from liberal arts and science and engineering universities reporting higher scores than those from medical universities. Additional differences in social media addiction were found according to only child status and parental educational levels (*P*<.001).

**Table 1.** Demographic characteristics and social media addiction scores (N=1766).

	Participants, n (%)	Social media addiction score (8-40), mean (SD)	Statistic		*P* value
			*t* test (*df*)	*F* test (*df*)	
Sex			0.27 (1764)	—^a^	.79
Male	857 (48.5)	21.05 (6.73)			
Female	909 (51.5)	21.13 (5.84)			
Age (y)			—	1.52 (2, 1763)	.22
18-19	839 (47.5)	20.87 (6.10)			
20-21	695 (39.4)	21.17 (6.44)			
22-24	232 (13.1)	21.66 (6.45)			
University type			—	50.91 (2, 1763)	<.001
Medical	793 (44.9)	19.53 (5.69)			
Science and engineering	252 (14.3)	21.39 (6.05)			
Liberal arts	721 (40.8)	22.7 (6.57)			
Academic year			—	0.29 (3, 1762)	.83
First year	895 (50.7)	21.01 (6.23)			
Second year	437 (24.7)	21.29 (6.17)			
Third year	325 (18.4)	21.14 (6.51)			
Fourth year or above	109 (6.2)	20.77 (6.57)			
Only child			3.98 (1764)	—	<.001
Yes	881 (49.9)	21.68 (6.14)			
No	885 (50.1)	20.5 (6.37)			
Father’s educational level			—	10.60 (2, 1763)	<.001
Primary school or lower	202 (11.4)	22.59 (6.41)			
Junior or senior high school	761 (43.1)	21.36 (5.94)			
College or higher	803 (45.5)	20.46 (6.49)			
Mother’s educational level			—	12.47 (2,1763)	<.001
Primary school or lower	284 (16.1)	22.32 (6.46)			
Middle school	787 (44.6)	21.38 (6.11)			
High school	695 (39.4)	20.26 (6.3)			

a Not applicable.

Table S1 in Multimedia Appendix 1 summarizes the descriptive statistics and correlations among parenting styles, perceived burdensomeness, thwarted belongingness, and social media addiction. Significant correlations were found between parenting styles and both perceived burdensomeness and thwarted belongingness (*P*<.001). Social media addiction was positively associated with parental rejection and overprotection but negatively associated with parental emotional warmth (*P*<.001). Furthermore, social media addiction was positively correlated with both perceived burdensomeness and thwarted belongingness (*P*<.001).

### Latent Profiles of Parenting Style

LPA was conducted using paternal and maternal rejection, emotional warmth, and overprotection as indicators (Table 2). Entropy values were high for both the 2- and 3-profile models (0.914‐0.933), indicating clear classification. Although the Akaike information criterion, BIC, and sample size–adjusted BIC values continued to decrease with additional profiles, model selection also considered interpretability. The 4- and 5-profile solutions mainly split existing profiles into smaller intermediate subgroups rather than identifying qualitatively distinct new configurations (Figure S1 in Multimedia Appendix 1). In contrast, the 3-profile solution identified 3 clear and theoretically meaningful parenting patterns with acceptable class proportions and the smallest class exceeding 10% of the sample. Taken together, the 3-profile model was selected as the optimal solution.

**Table 2.** Model fit indexes of latent profile analysis using short-form Egna Minnen av Barndoms Uppfostran for Chinese scores.

Profile model	AIC^a^	BIC^b^	aBIC^c^	Entropy	BLRT^d^	Proportions^e^
1	63,088	63,154	63,115	1	—^f^	1
2	60,507	60,611	60,551	0.933	0.010	0.732/0.268
3	59,434	59,576	59,494	0.914	0.010	0.619/0.109/0.272
4	58,968	59,149	59,044	0.848	0.010	0.370/0.297/0.260/0.072
5	57,756	57,975	57,848	0.884	0.010	0.353/0.293/0.228/0.066/0.061

a AIC: Akaike information criterion.

b BIC: Bayesian information criterion.

c aBIC: sample size–adjusted BIC.

d BLRT: bootstrap likelihood ratio test.

e Proportions indicate the proportion of each profile in the model.

f Not applicable.

**Figure 1.**
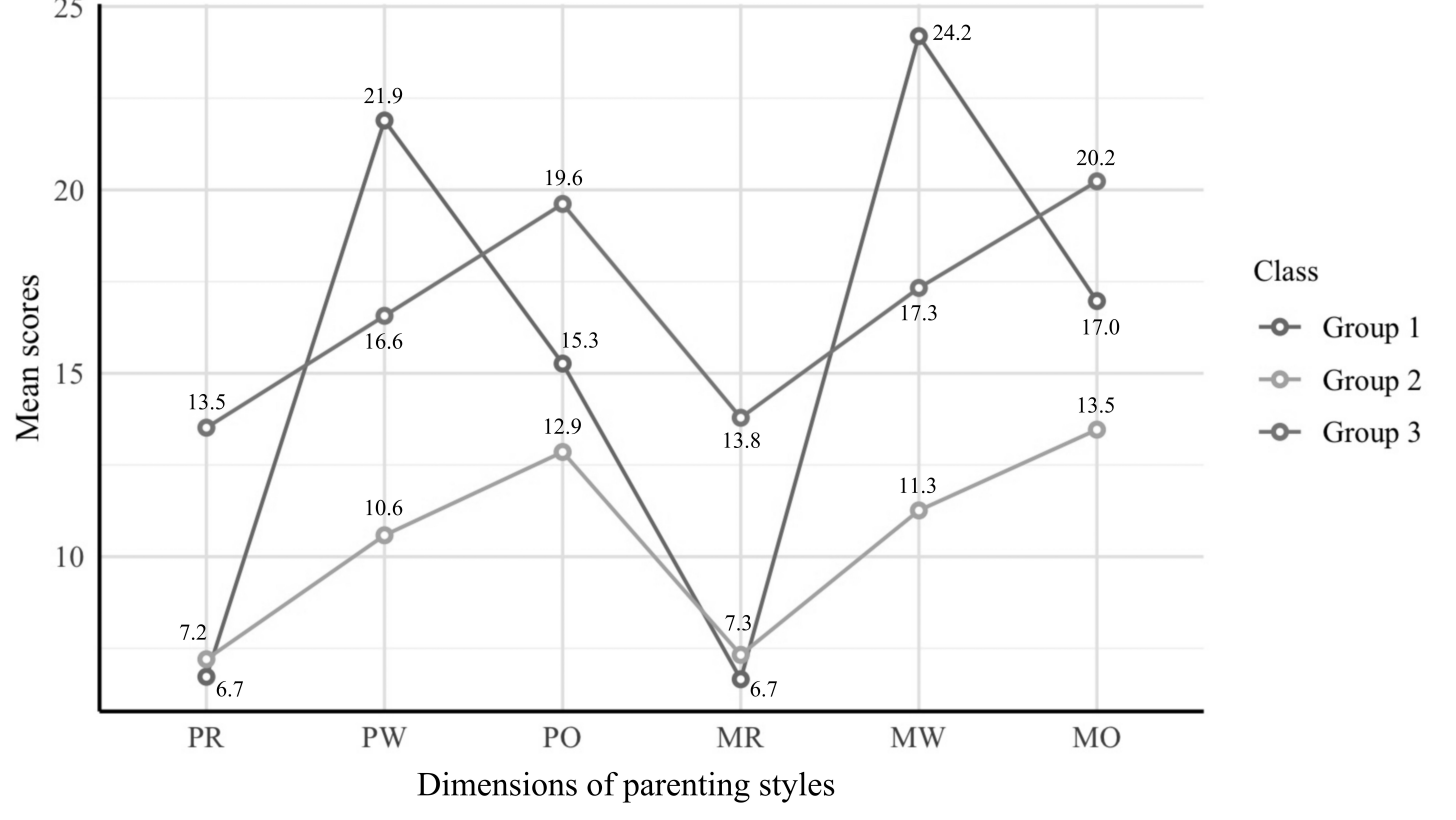
Latent profiles of parenting styles: mean short-form Egna Minnen av Barndoms Uppfostran for Chinese scores across classes. Group 1: supportive parenting (1094/1766, 61.9%); group 2: emotionally distant parenting (192/1766, 10.9%); group 3: controlling-critical parenting (480/1766, 27.2%); MO: maternal overprotection; MR: maternal rejection; MW: maternal emotional warmth; PO: paternal overprotection; PR: paternal rejection; PW: paternal emotional warmth.

Figure 1 shows the mean S-EMBU-C scores across the 3 latent parenting profiles. Group 1, labeled the supportive parenting group (1094/1766, 61.9%), represented the majority and was characterized by high parental emotional warmth and moderate levels of rejection and overprotection. Group 2, the emotionally distant parenting group (192/1766, 10.9%), was marked by lower parental emotional warmth. Group 3, the controlling-critical parenting group (480/1766, 27.2%), showed elevated parental rejection and overprotection. Significant differences were observed across all parenting profiles (*P*<.001). Parental rejection and overprotection were highest in the controlling-critical parenting group compared to the other 2 groups (*P*<.001), whereas parental emotional warmth was highest in the supportive parenting group (*P*<.001; Table S2 in Multimedia Appendix 1).

The emotionally distant parenting group was relatively small, which may have reduced power to detect small effects. Therefore, nonsignificant subgroup findings should be interpreted with caution.

### Hierarchical Regression Analyses of Social Media Addiction

Table 3 presents the results of the hierarchical regression analyses. In model 1, parenting profiles significantly predicted social media addiction. Compared with the supportive parenting group, participants in the emotionally distant (β=1.417; *P*=.003) and controlling-critical (β=4.438; *P*<.001) parenting groups reported higher levels of social media addiction. After adjusting for demographic variables in model 2, these associations remained significant. In model 3, both perceived burdensomeness (β=0.096; *P*<.001) and thwarted belongingness (β=0.089; *P*<.001) were positively associated with social media addiction. Importantly, even after accounting for these interpersonal need factors, the controlling-critical parenting group continued to report higher levels compared with the supportive parenting group (β=2.081; *P*<.001).

**Table 3.** Regression analyses predicting social media addiction from parenting profiles.

Variables	Model 1^a^		Model 2^b^		Model 3^c^	
	β (SE)	*P* value	β (SE)	*P* value	β (SE)	*P* value
Parenting profile						
Supportive parenting	Reference	Reference	Reference	Reference	Reference	Reference
Emotionally distant parenting	1.417 (0.468)	.003	0.774 (0.478)	.11	–0.419 (0.498)	.40
Controlling-critical parenting	4.438 (0.328)	<.001	3.814 (0.347)	<.001	2.081 (0.400)	<.001
Sex						
Male	—^d^	—	Reference	Reference	Reference	Reference
Female	—	—	0.740 (0.287)	.01	0.865 (0.282)	.002
Age	—	—	−0.098 (0.121)	.42	−0.148 (0.119)	.22
University type						
Medical	—	—	Reference	Reference	Reference	Reference
Science and engineering	—	—	1.641 (0.441)	<.001	1.397 (0.433)	.001
Liberal arts	—	—	2.187 (0.343)	<.001	1.823 (0.339)	<.001
Academic year						
First year	—	—	Reference	Reference	Reference	Reference
Second year	—	—	0.133 (0.379)	.73	0.259 (0.372)	.49
Third year	—	—	0.795 (0.457)	.08	0.862 (0.448)	.05
Fourth year or above	—	—	−0.231 (0.697)	.74	−0.173 (0.684)	.80
Only child						
Yes	—	—	Reference	Reference	Reference	Reference
No	—	—	−0.579 (0.311)	.06	−0.681 (0.305)	.03
Father’s educational level						
Primary school or lower	—	—	Reference	Reference	Reference	Reference
Junior or senior high school	—	—	−0.421 (0.514)	.41	−0.407 (0.504)	.42
College or above	—	—	−0.090 (0.593)	.88	−0.064 (0.582)	.91
Mother’s educational level						
Primary school or lower	—	—	Reference	Reference	Reference	Reference
Middle school	—	—	−0.019 (0.461)	.97	0.176 (0.453)	.70
High school	—	—	−0.314 (0.562)	.58	0.008 (0.552)	.99
Perceived burdensomeness	—	—	—	—	0.096 (0.022)	<.001
Thwarted belongingness	—	—	—	—	0.089 (0.015)	<.001

a *R*2=0.094.

b *R*2=0.125.

c *R*2=0.160.

d Not applicable.

### Pathways Linking Parenting Profiles to Social Media Addiction

**Figure 2.**
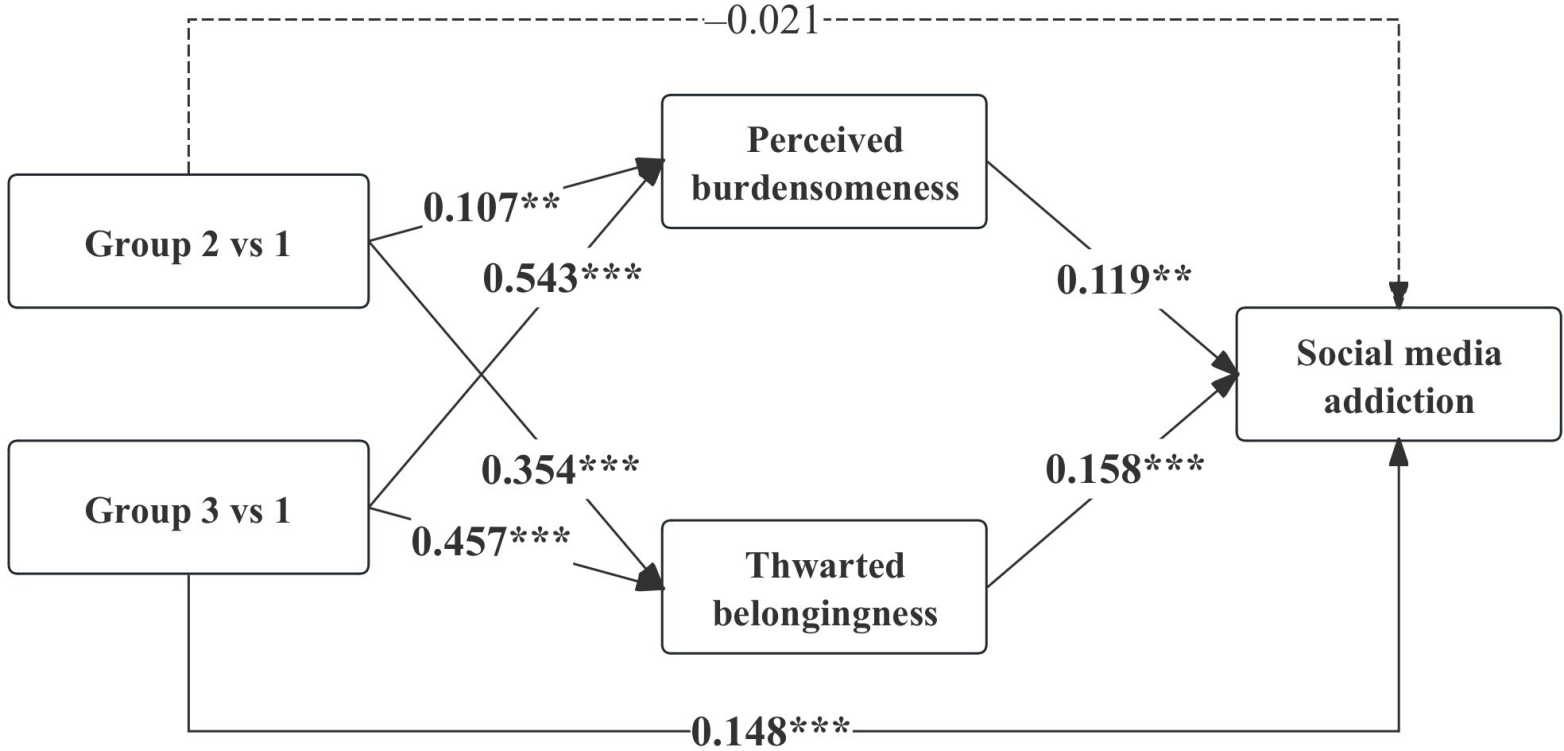
Mediation model from parenting profiles to social media addiction through perceived burdensomeness and thwarted belongingness. Standardized coefficients (β) are reported. ***P<.001; **P<.01; *P<.05; group 1: supportive parenting; group 2: emotionally distant parenting; group 3: controlling-critical parenting.

Figure 2 presents the pathways linking parenting profiles to social media addiction through perceived burdensomeness and thwarted belongingness. Model fit indexes indicated a good fit (comparative fit index=0.963; Tucker-Lewis index=0.931; root mean squared error of approximation=0.038; standardized root mean squared residual=0.017).

Parenting profiles were significantly associated with both interpersonal need dimensions. Compared with the supportive parenting group, participants in the emotionally distant parenting group reported higher levels of perceived burdensomeness (β=0.107; *P*<.001) and thwarted belongingness (β=0.354; *P*<.001). Similarly, students in the controlling-critical parenting group showed positive associations with perceived burdensomeness (β=0.543; *P*<.001) and thwarted belongingness (β=0.457; *P*<.001). In turn, both perceived burdensomeness (β=0.119; *P*=.001) and thwarted belongingness (β=0.158; *P*<.001) were positively associated with social media addiction. Detailed estimates of all paths in the model can be found in Table S3 in Multimedia Appendix 1.

Mediation analyses further indicated that the total indirect effect was significant for both the emotionally distant (β=0.069; *P*<.001) and controlling-critical (β=0.137; *P*<.001) parenting groups compared with the supportive parenting group. Notably, the direct effect of the emotionally distant parenting group was not significant (β=–0.021; *P*=.43), whereas the controlling-critical parenting group showed a significant direct effect on social media addiction (β=0.148; *P*<.001).

### Multigroup Analyses by Sex and Only Child Status

**Figure 3.**
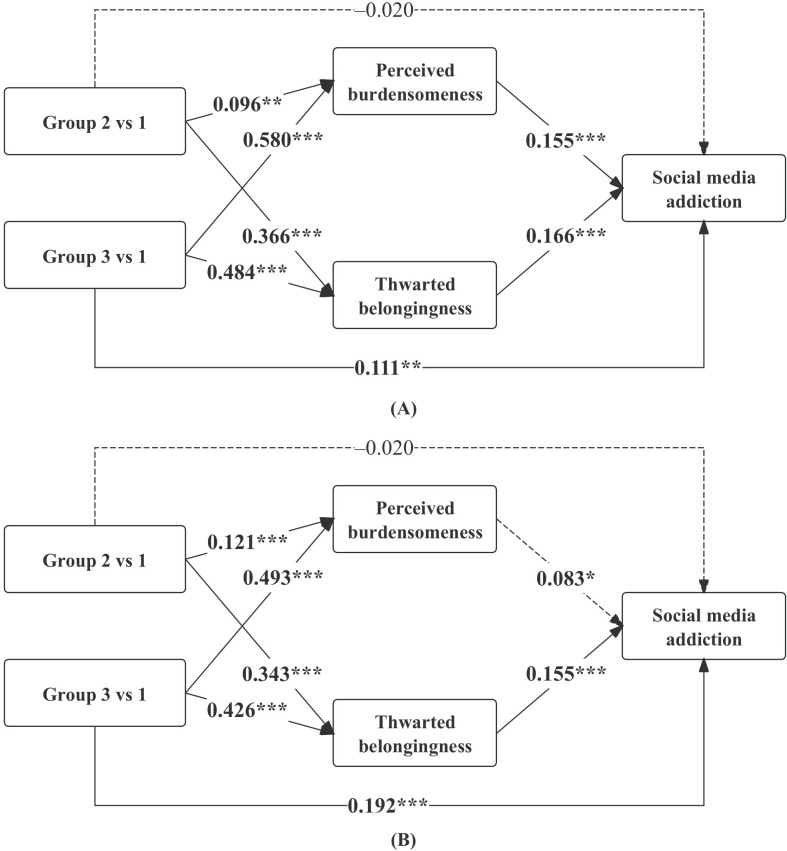
Multigroup analyses stratified by sex. Standardized coefficients (β) are reported for male individuals (A) and female individuals (B). ***P<.001; **P<.01; *P<.05; group 1: supportive parenting; group 2: emotionally distant parenting; group 3: controlling-critical parenting.

Multigroup analyses revealed that the association patterns between parenting profiles and the mediating variables were largely consistent across subgroups. Thwarted belongingness was positively associated with social media addiction in all groups, including male students (β=0.150; *P*<.001), female students (β=0.175; *P*<.001), only children (β=0.166; *P*<.001), and non–only children (β=0.155; *P*<.001). In contrast, perceived burdensomeness was significantly associated with social media addiction among male students (β=0.180; *P*<.001) but not among female students (β=0.038; *P*=.30). For only child status, perceived burdensomeness remained significant in both only children (β=0.155; *P*<.001) and non–only children (β=0.083; *P*=.02). In addition, the direct effect of the controlling-critical parenting profile on social media addiction remained significant across subgroups, including male students (β=0.147; *P*<.001), female students (β=0.158; *P*<.001), only children (β=0.111; *P*=.009), and non–only children (β=0.192; *P*<.001). The results of multigroup analyses based on sex and only child status are shown in Figures3  4.

**Figure 4.**
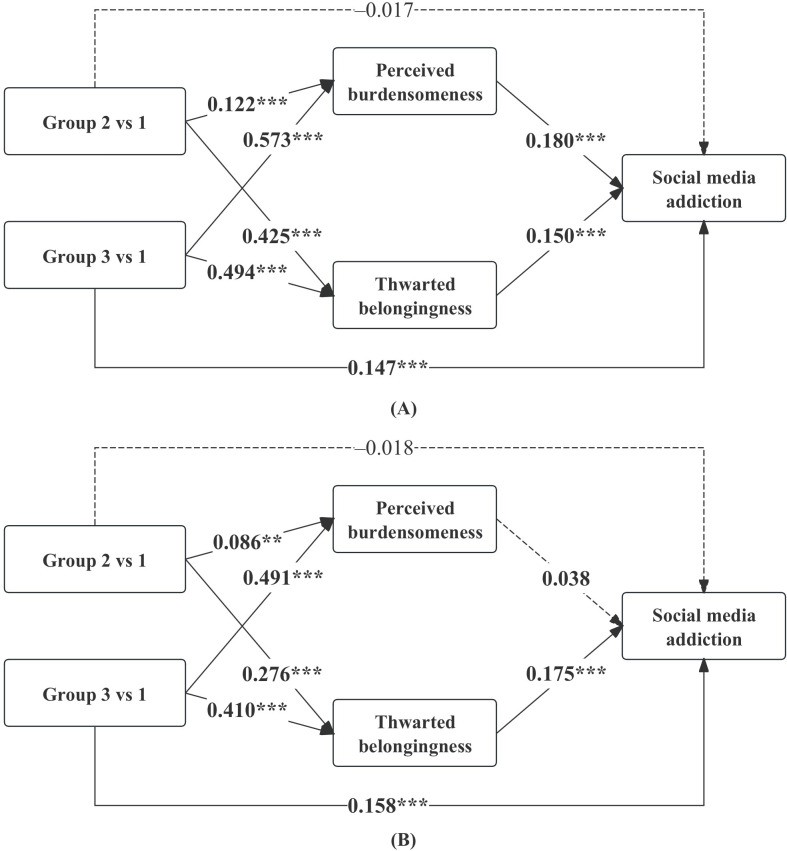
Multigroup analyses stratified by only child status. Standardized coefficients (β) are reported for only children (A) and non–only children (B). ***P<.001; **P<.01; *P<.05; group 1: supportive parenting; group 2: emotionally distant parenting; group 3: controlling-critical parenting.

## Discussion

### Main Findings

This study identified 3 latent profiles of parenting styles among university students: supportive, emotionally distant, and controlling-critical parenting. Compared to the supportive group, both the emotionally distant and controlling-critical groups reported higher levels of social media addiction, a prominent form of digital dependence. These associations were partly mediated by perceived burdensomeness and thwarted belongingness.

Drawing on paternal and maternal rejection, emotional warmth, and overprotection, 3 parenting profiles were identified through LPA. Consistent with previous studies on Chinese families, the largest profile was supportive parenting, characterized by higher levels of emotional warmth and lower levels of control [30,31]. Beyond this parenting style, 2 maladaptive profiles also emerged: an emotionally distant group with limited emotional warmth and a controlling-critical group with elevated rejection and overprotection. Unlike earlier studies that broadly categorized parenting into positive and negative types [32], our findings distinguished 2 qualitatively different forms of maladaptive parenting, with one defined by deficiencies in positive parenting and the other defined by excessive negative behaviors. This distinction provides a foundation for further understanding how parenting styles contribute to different health risks.

In this study, university students with emotionally distant or controlling-critical parents had higher levels of social media addiction compared to those with supportive parents. Prior studies have indicated that parental rejection and overprotection increase the risk of digital addiction [33,34], whereas a supportive general parenting context provides protection against problematic engagement with social media [35,36]. Researchers have further suggested that maladaptive parenting may undermine children’s 3 fundamental psychological needs, namely, relatedness, autonomy, and competence, which subsequently drives individuals to seek compensation through online interactions [37]. In line with this perspective, the attenuation of the association between parenting and social media addiction after accounting for psychological factors highlights the critical role of perceived burdensomeness and thwarted belongingness.

Path analysis further confirmed that perceived burdensomeness and thwarted belongingness mediated the association between parenting profiles and social media addiction. Previous research indicates that maladaptive parenting can contribute to heightened psychological and cognitive vulnerabilities [38,39], which in turn foster unmet interpersonal needs [22]. Although originally conceptualized within the framework of suicidal behavior [16], perceived burdensomeness and thwarted belongingness have been linked to a broader range of adverse outcomes [17,19,40]. Specifically, perceived burdensomeness has been associated with social media addiction [21], suggesting that it may represent an internal manifestation of unsatisfied social needs in offline interactions. To alleviate this deficiency, individuals may increasingly rely on digital technologies as a compensatory mechanism, increasing the risk of problematic use. Building on prior evidence, our findings indicate perceived burdensomeness and thwarted belongingness as psychological pathways linking maladaptive parenting to social media addiction.

The association between parenting and unmet interpersonal needs may vary across different parenting profiles. Previous research suggests that specific parenting styles are linked to distinct personality traits [41], which may subsequently shape psychological states. Consistently, we found that emotionally distant parenting was more strongly related to thwarted belongingness, reflecting insufficient emotional responsiveness that fosters insecure attachment and undermines children’s sense of connection and inclusion [42]. In contrast, controlling and critical parenting often involves disapproval of children’s feelings and excessive control that can foster perceptions of being a burden to others [37] and, thus, showed a closer association with perceived burdensomeness.

This heterogeneity was further reflected in the different mediation patterns observed across the 2 maladaptive parenting profiles. For the emotionally distant profile, the association with social media addiction became nonsignificant after perceived burdensomeness and thwarted belongingness were included. This pattern suggests that emotionally distant parenting may influence social media addiction primarily through unmet interpersonal needs, particularly reduced belongingness. In contrast, the controlling-critical profile showed evidence of partial mediation. One possible explanation is that, beyond undermining interpersonal needs, controlling-critical parenting may also affect other psychological processes, such as perceived meaning and social support [37,43], which were not captured in this model. Together, these findings indicate that different forms of maladaptive parenting may contribute to social media addiction through partially overlapping yet distinct psychological pathways.

In the multigroup analyses, perceived burdensomeness was associated with social media addiction only among male students, whereas thwarted belongingness remained significant across subgroups. Compared with thwarted belongingness, perceived burdensomeness is more closely related to self-worth and perceived social value [16]. In many sociocultural contexts, male individuals may be more likely to tie self-evaluation to competence, independence, and usefulness. Under such conditions, feeling like a burden to others may more readily translate into compensatory engagement with social media, where recognition or distraction can be more easily obtained. This interpretation is consistent with prior studies suggesting gender differences in the roles of unmet interpersonal needs as risk factors, with perceived burdensomeness appearing particularly relevant for male individuals [44]. In contrast, the stable association with thwarted belongingness across subgroups suggests that unmet needs for social connection may represent a more general pathway to problematic social media use.

Potential conceptual overlap between parenting styles and unmet interpersonal needs should be acknowledged. However, these constructs differ in temporal focus and conceptual level, with parenting reflecting early-life experiences and interpersonal needs capturing current internal states. Therefore, these needs may represent internalized consequences of earlier experiences rather than direct equivalents of parenting. Although shared method variance and partial overlap cannot be ruled out, the findings offer several theoretical contributions. They extend the interpersonal theory of suicide to the domain of digital addiction and integrate it with the social compensation hypothesis, highlighting that compensatory online engagement may operate through specific unmet interpersonal needs. The identification of distinct parenting profiles further shows that different forms of maladaptive parenting may confer risk through distinct psychological pathways. These findings provide a more cohesive framework for understanding how early family experiences shape vulnerability to problematic social media use in emerging adulthood.

### Implications

These findings reveal the potential role of early family experiences in the development of social media addiction during emerging adulthood, underscoring the importance of supportive parenting practices. Emotional warmth can be fostered through expressions of care, positive interactions, and open communication [45]. Meanwhile, parental supervision should be aligned with developmental needs, respecting children’s autonomy and personal boundaries while minimizing psychological control [45]. Furthermore, unmet interpersonal needs may represent a fundamental psychological mechanism underlying social media addiction. These states are modifiable through interventions that strengthen interpersonal communication skills and restore self-worth, thereby reducing reliance on online interactions as a compensatory strategy.

The distinct mediation patterns observed across parenting profiles suggest that intervention priorities may differ by family context. For individuals from emotionally distant families, interventions focused on strengthening belongingness and emotional connectedness may be particularly relevant. In contrast, those from controlling-critical families may require broader multicomponent approaches beyond interpersonal needs while still emphasizing the reduction of perceived burdensomeness through greater autonomy support. In addition, approaches may benefit from sensitivity to gender differences, particularly in addressing burdensomeness-related cognitions and self-worth among male students. These findings suggest that screening for parenting-related risk patterns and unmet interpersonal needs may help guide more tailored interventions.

### Limitations

This study has several limitations that should be acknowledged. First, the cross-sectional design prevents causal inference, and the observed associations may be influenced by bidirectional or reciprocal effects. Future longitudinal or experimental studies are warranted to clarify the temporal relationship among parenting styles, interpersonal needs, and social media addiction. Second, parenting styles were assessed through participants’ retrospective self-reports, which may be subject to recall bias and personal interpretation. Third, although the study examined social media addiction, it did not differentiate between specific platforms or investigate the underlying motives for social media use, which may provide further insights into patterns of digital engagement. Fourth, the study sample was restricted to university students in Shanghai, which may limit the generalizability of the findings to other regions of China and other cultural contexts. In particular, the salience of family interdependence, relational obligations, and unmet interpersonal needs may differ across sociocultural settings. Therefore, cross-cultural studies are needed to examine whether similar parenting profiles and interpersonal pathways operate in other contexts. Fifth, although a multistage sampling approach was used to account for variation across universities, majors, and academic years, the sample cannot be considered fully representative of university students in Shanghai because proportional allocation across strata was not implemented. In addition, because demographic information for the excluded respondents was not retained or was not comparable, potential selection bias cannot be ruled out. Exclusion criteria may also have preferentially excluded less attentive or lower-engagement respondents. Sixth, although the overall sample size was relatively large, some subgroup analyses were based on smaller subsamples, particularly the emotionally distant parenting group and the stratified multigroup models. Accordingly, nonsignificant findings in these analyses should be interpreted cautiously. Seventh, although intergenerational coresidence is relatively common in the Chinese context and may influence relational experiences, this specific aspect of family structure was not assessed in this study. Future research should incorporate measures of intergenerational context to better understand its influence on social media use. Finally, this study did not account for potentially relevant confounders such as mental health problems or personality traits, which may have influenced both interpersonal needs and social media use.

### Conclusions

This study identified 3 distinct parenting profiles among university students, namely, supportive, emotionally distant, and controlling-critical parenting, and examined their associations with social media addiction. Students with emotionally distant or controlling-critical parents reported higher levels of social media addiction compared to those with supportive parents. Perceived burdensomeness and thwarted belongingness mediated these associations. Notably, perceived burdensomeness was associated with social media addiction only among male students, highlighting potential gender differences in underlying mechanisms. These findings underscore the importance of early interventions that promote parental emotional warmth, reduce rejecting and controlling behaviors, and address unmet interpersonal needs by strengthening offline social connections. Prevention efforts may further benefit from tailored approaches that consider specific interpersonal vulnerabilities and family contexts. The ultimate goal is to mitigate the risk of social media addiction among young adults.

## Supplementary material

10.2196/91861 Multimedia Appendix 1 Additional tables and figures supporting the main analyses.

10.2196/91861 Checklist 1 STROBE checklist.

## Abbreviations

BIC: Bayesian information criterion
INQ-15: 15-item version of the Interpersonal Needs Questionnaire
LPA: latent profile analysis
S-EMBU-C: short-form Egna Minnen av Barndoms Uppfostran for Chinese
