# Supplementary material for: The Mediating Role of Interpersonal Needs in Perceived Parenting Styles and Social Media Addiction Among University Students: Cross-Sectional Study
Source: J Med Internet Res. 2026 May 27;28:e91861. doi: 10.2196/91861 (PMC13215577; doi:10.2196/91861)
Supplement: Multimedia Appendix 1 [file jmir-v28-e91861-s001.docx]

**Table S1. Descriptive Statistics and Correlations Among parenting styles, perceived burdensomeness, thwarted belongingness, and social media addiction**

| **Variables** | **M ± SD** | **Range** | **1** | **2** | **3** | **4** | **5** | **6** | **7** | **8** | **9** |
| --- | --- | --- | --- | --- | --- | --- | --- | --- | --- | --- | --- |
| 1. **Paternal rejection** | 8.62 ± 3.89 | 6 – 24 | — |  |  |  |  |  |  |  |  |
| 1. Paternal emotional warmth | 19.21 ± 6.00 | 7 – 28 | -0.219^***^ | — |  |  |  |  |  |  |  |
| 1. **Paternal overprotection** | 16.18 ± 4.45 | 8 – 32 | 0.495^***^ | 0.057^*^ | — |  |  |  |  |  |  |
| 1. **Maternal rejection** | 8.67 ± 3.87 | 6 – 24 | 0.695^***^ | -0.185^***^ | 0.480^***^ | — |  |  |  |  |  |
| 1. Maternal emotional warmth | 20.92 ± 5.99 | 7 – 28 | -0.241^***^ | 0.693^***^ | -0.036 | -0.295^***^ | — |  |  |  |  |
| 1. **Maternal overprotection** | 17.48 ± 4.73 | 8 – 32 | 0.298^***^ | 0.049^***^ | 0.58^***^ | 0.475^***^ | 0.077^***^ | — |  |  |  |
| 1. Perceived burdensomeness | 11.32 ± 7.74 | 6 – 42 | 0.539^***^ | -0.237^***^ | 0.324^***^ | 0.525^***^ | -0.303^***^ | 0.216^***^ | — |  |  |
| 1. Thwarted belongingness | 27.12 ± 11.06 | 9 – 61 | 0.329^***^ | -0.508^***^ | 0.188^***^ | 0.350^***^ | -0.527^***^ | 0.101^***^ | 0.445^***^ | — |  |
| 1. Social media addiction | 21.09 ± 6.29 | 8 – 40 | 0.306^***^ | -0.160^***^ | 0.260^***^ | 0.315^***^ | -0.195^***^ | 0.233*** | 0.291^***^ | 0.287^***^ | — |

Note: M = Mean; SD = Standard Deviation. Values in parentheses on the diagonal are Cronbach's alpha coefficients.

*p < .05, **p < .01, ***p < .001

**Table S2. Mean differences in parenting styles across latent parenting profiles**

| **Variables** | **Group 1** | **Group 2** | **Group 3** | ANOVA | | | |
| --- | --- | --- | --- | --- | --- | --- | --- |
|  |  |  |  | F | Overall *P*-value | *P*-value (1 vs 2) | *P*-value (1 vs 3) |
| **Paternal rejection** | 6.73±1.46 | 7.20±2.19 | 13.52±4.00 | 1277.19 | < 0.001 | 0.043 | < 0.001 |
| Paternal emotional warmth | 21.89±4.98 | 10.58±3.78 | 16.57±4.16 | 589.67 | < 0.001 | < 0.001 | < 0.001 |
| **Paternal overprotection** | 15.26±4.01 | 12.85±2.98 | 19.62±3.82 | 292.93 | < 0.001 | < 0.001 | < 0.001 |
| **Maternal rejection** | 6.66±1.37 | 7.32±2.09 | 13.79±3.61 | 1675.51 | < 0.001 | 0.001 | < 0.001 |
| Maternal emotional warmth | 24.19±3.82 | 11.26±4.38 | 17.33±4.07 | 1144.63 | < 0.001 | < 0.001 | < 0.001 |
| **Maternal overprotection** | 16.97±4.54 | 13.46±3.27 | 20.23±4.07 | 190.48 | < 0.001 | < 0.001 | < 0.001 |

Note: S-EMBU-C scores are presented as Mean ± SD

**Table S3. Bootstrapped effects of parenting profiles on social networking addiction via perceived burdensomeness and thwarted belongingness**

| Path | Coefficients | S.E. | *P*-value | 95%CI | Standardized coefficients |
| --- | --- | --- | --- | --- | --- |
| **Perceived burdensomeness ~** |  |  |  |  |  |
| **Parenting profile** |  |  |  |  |  |
| Supportive parenting | Ref | | | | |
| Emotionally distant parenting | 2.651 | 0.532 | < 0.001 | 1.619—3.705 | 0.107 |
| Controlling and critical parenting | 9.452 | 0.449 | < 0.001 | 8.581—10.331 | 0.543 |
| **Thwarted belongingness~** |  |  |  |  |  |
| **Parenting profile** |  |  |  |  |  |
| Supportive parenting | Ref | | | | |
| Emotionally distant parenting | 12.566 | 0.791 | < 0.001 | 10.978—14.062 | 0.354 |
| Controlling and critical parenting | 11.366 | 0.480 | < 0.001 | 10.433—12.298 | 0.457 |
| **Social media addiction~** |  |  |  |  |  |
| **Parenting profile** |  |  |  |  |  |
| Supportive parenting | Ref | | | | |
| Emotionally distant parenting | -0.419 | 0.534 | 0.432 | -1.466—0.622 | -0.021 |
| Controlling and critical parenting | 2.081 | 0.423 | < 0.001 | 1.243—2.910 | 0.148 |
| **Perceived burdensomeness~** | 0.096 | 0.028 | 0.001 | 0.038—0.151 | 0.119 |
| **Thwarted belongingness** | 0.089 | 0.018 | < 0.001 | 0.056—0.124 | 0.158 |
| **Sex** | 0.865 | 0.281 | 0.002 | 0.308—1.422 | 0.069 |
| **Age** | -0.148 | 0.135 | 0.266 | -0.420—0.115 | -0.036 |
| **Major** |  |  |  |  |  |
| Medical | Ref | | | | |
| Science/engineering | 1.397 | 0.428 | 0.001 | 0.569—2.259 | 0.078 |
| Liberal arts | 1.823 | 0.343 | < 0.001 | 1.146—2.479 | 0.143 |
| **Academic year** |  |  |  |  |  |
| First year | Ref | | | | |
| Second year | 0.259 | 0.379 | 0.494 | -0.499—0.985 | 0.018 |
| Third year | 0.862 | 0.466 | 0.064 | -0.039—1.785 | 0.053 |
| Fourth/Fifth year | -0.173 | 0.757 | 0.819 | -1.624—1.320 | -0.007 |
| **Single** |  |  |  |  |  |
| Yes | Ref | | | | |
| No | -0.681 | 0.298 | 0.022 | -1.278—-0.110 | -0.054 |
| ****Father's education**** |  |  |  |  |  |
| Primary school or below | Ref | | | | |
| Junior/senior high school | -0.407 | 0.496 | 0.412 | -1.367—0.579 | -0.032 |
| College or above | -0.064 | 0.583 | 0.912 | -1.239—1.081 | -0.005 |
| ****Mother's education**** |  |  |  |  |  |
| Primary school or below | Ref | | | | |
| High school | 0.176 | 0.444 | 0.692 | -0.696—1.056 | 0.014 |
| College or above | 0.008 | 0.538 | 0.988 | -1.048—1.048 | 0.001 |

**Figure S1. Latent profiles of parenting styles: mean scores of S-EMBU-C across classes**

**
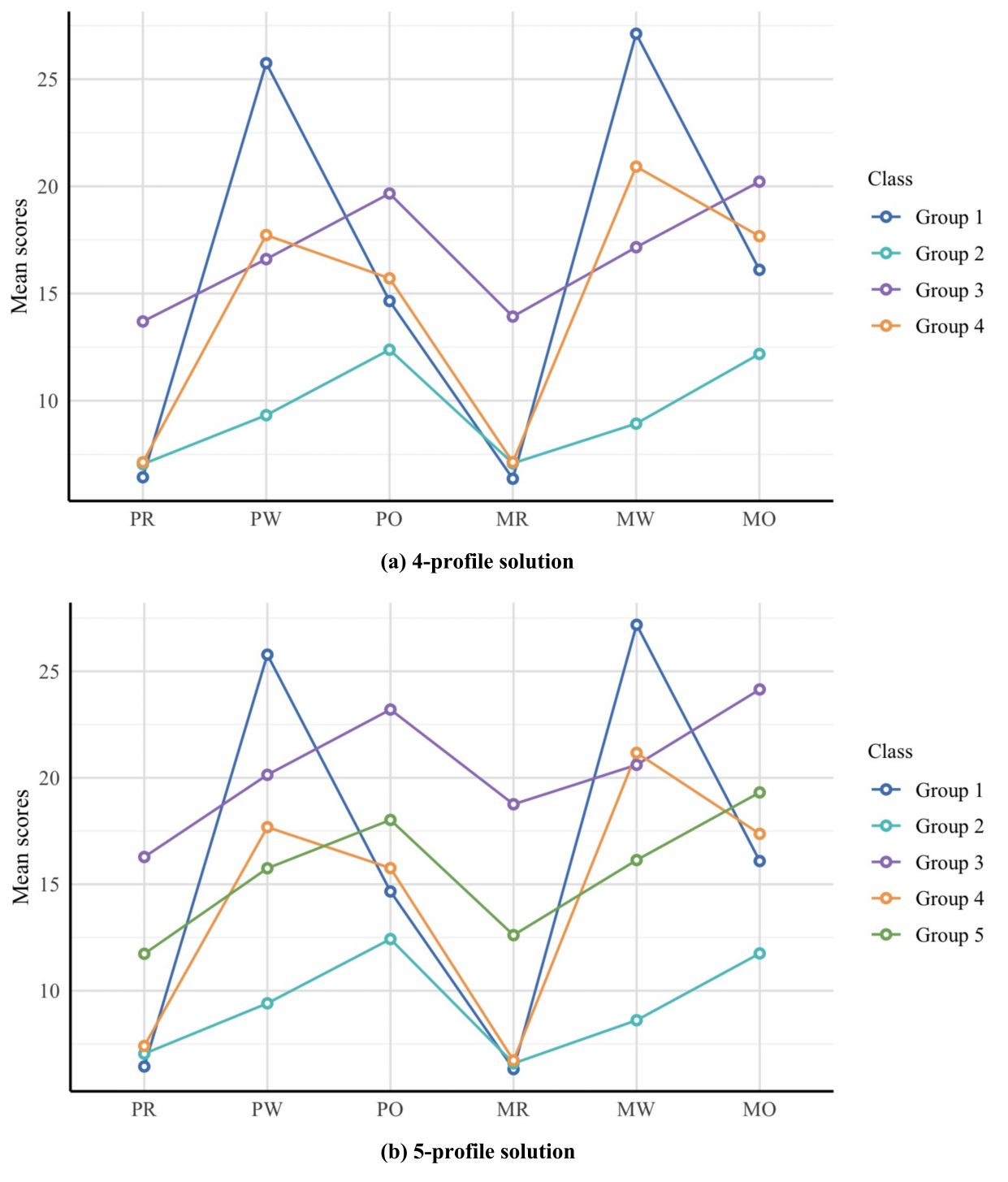
**

PR: Paternal rejection; PW: Paternal emotional warmth; PO: Paternal overprotection; MR: Maternal rejection; MW: Maternal emotional warmth; MO: Maternal overprotection.
